# Is There a Need for Sex‐Tailored Lipoprotein(a) Cut‐Off Values for Coronary Artery Disease Risk Stratification?

**DOI:** 10.1002/clc.70012

**Published:** 2024-09-12

**Authors:** Ece Yurtseven, Dilek Ural, Erol Gursoy, Bekay Omer Cunedioglu, Orhan Ulas Guler, Kemal Baysal, Saide Aytekin, Vedat Aytekin, Meral Kayikcioglu

**Affiliations:** ^1^ Department of Cardiology Koc University School of Medicine Istanbul Turkey; ^2^ Department of Biochemistry Koc University School of Medicine Istanbul Turkey; ^3^ Department of Cardiology Ege University School of Medicine Izmir Turkey

**Keywords:** cardiovascular disease risk, coronary artery disease, lipoprotein(a), sex, women and men

## Abstract

**Background:**

Lipoprotein(a) [Lp(a)] plasma level is a well‐known risk factor for coronary artery disease (CAD). Existing data regarding the influence of sex on the Lp(a)‐CAD relationship are inconsistent.

**Objective:**

To investigate the relationship between Lp(a) and CAD in men and women and to elucidate any sex‐specific differences that may exist.

**Methods:**

Data of patients with Lp(a) measurements who were admitted to a tertiary university hospital, Koc University Hospital, were analyzed. The relationship between Lp(a) levels and CAD was explored in all patients and in subgroups created by sex. Two commonly accepted Lp(a) thresholds ≥ 30 and ≥ 50 mg/dL were analyzed.

**Results:**

A total of 1858 patients (mean age 54 ± 17 years; 53.33% females) were included in the analysis. Lp(a) was an independent predictor of CAD according to the multivariate regression model for the entire cohort. In all cohort, both cut‐off values (≥ 30 and ≥ 50 mg/dL) were detected as independent predictors of CAD (*p* < 0.001). In sex‐specific analysis, an Lp(a) ≥ 30 mg/dL was an independent predictor of CAD only in women (*p* < 0.001), but Lp(a) ≥ 50 mg/dL was a CAD predictor both in men and women (men, *p* = 0.004; women, *p* = 0.047).

**Conclusion:**

The findings of this study may suggest that different thresholds of Lp(a) level can be employed for risk stratification in women compared to men.

## 1 Introduction

Lipoprotein(a) [Lp(a)] was recognized as a macromolecule in plasma in 1963 [1]. It is a lipoprotein similar to low‐density lipoprotein (LDL), which consists of apolipoprotein B100 and cholesterol. It differs from LDL by containing apolipoprotein a [Apo(a)], which is bound to the molecule covalently by a disulfide bond. Apo(a) can bind to extracellular matrix proteins, leading to subendothelial cholesterol accumulation [2]. Accumulation of oxidized phospholipids, which prefer to bind Apo(a), stimulates the inflammatory response and enhances atherosclerotic vascular changes [3].

Many clinical studies have verified high Lp(a) level as a risk factor for coronary artery disease (CAD), stroke, and aortic stenosis [4]. Recent guidelines recommend at least a one‐time measurement of Lp(a) level in adult life to find individuals with very high Lp(a) levels or measurement of Lp(a) level in patients with a family history of premature cardiovascular disease (CVD). According to metanalyses, the association of Lp(a) level and CVD risk is continuous and unrelated to other lipoprotein levels and traditional risk factors [5, 6]. Nevertheless, for the purpose of simplicity, certain cut‐off values have been determined to describe Lp(a) associated risk in clinical practice. For instance, the American College of Cardiology/American Heart Association (ACCF/AHA) guidelines and the Canadian Cardiovascular Society (CCS) guidelines identify an Lp(a) level of ≥ 50 mg/dL as indicative of increased risk [7, 8]. The European Atherosclerosis Society (EAS) Consensus Document considers an Lp(a) level of ≥ 30 mg/dL as abnormal, while the European Society of Cardiology (ESC) recommends a one‐time Lp(a) measurement in an individual's lifetime to detect levels exceeding 180 mg/dL [9, 10].

The plasma level of Lp(a) is mainly determined by genetic factors [11]. Many studies have revealed that the relationship between Lp(a) level and CVD risk is not significant in some cohorts. For instance, in Africans and Arabs, the Lp(a) level was not helpful in predicting CVD [12]. Sex also influences Lp(a) levels; Lp(a) levels are typically higher in women, particularly during pregnancy and menopause, and are affected by hormone replacement therapy—trends not observed in men [13, 14]. Despite the knowledge of discrepancies in Lp(a) levels in men and women, there is inconsistency in the results of the studies investigating the sex differences in the relationship between Lp(a) levels and CVD [15, 16]. The contribution of Lp(a) to the stratification of CAD risk, the primary component of CVD, may exhibit sex‐specific variations, underscoring the need for distinct assessment approaches in men and women.

Therefore, in this study, we aimed (1) to assess the relationship between Lp(a) levels and CAD according to sex and (2) to evaluate the appropriateness of commonly used cut‐off levels in determining the risk in both sexes.

## 2 Methods

The study has been carried out in accordance with the Declaration of Helsinki and was authorized by the Local Ethics Committee of Koc University. Data from patients who attended the cardiology and internal medicine outpatient clinics at Koc University Hospital with nonurgent complaints between November 2015 and November 2020 and who had recorded Lp(a) levels were analyzed. Data from inpatient care and emergency department patients were not included in the analysis; therefore, patients admitted during acute settings were excluded. On a routine basis, Lp(a) measurements are conducted in patients with hyperlipidemia or suspected hyperlipidemia, in patients with CAD, diabetes mellitus, or those at risk for CVD, in accordance with health insurance regulations and clinician decision pathways in our institution.

During the specified period, 122 620 patients were admitted to the cardiology and internal medicine outpatient clinics, 3919 of whom had an Lp(a) level measurement. Acute and chronic inflammation might influence Lp(a) levels, possibly due to the IL‐6 response element in the *LPA* gene [17]. Consequently, we excluded patients with conditions associated with inflammation, including liver disease, severe kidney disease, and malignancy. Additionally, since hormone replacement therapy can modify Lp(a) levels and type 1 diabetes presents a distinct relationship with CAD, individuals with these conditions were also excluded from the study [14]. After excluding 2061 patients based on the exclusion criteria mentioned below, data from 1858 patients were ultimately analyzed. Patients younger than 18 years (*n* = 29), those with type 1 diabetes mellitus (*n* = 21), acute infection and/or inflammation (patients who had high sensitive C reactive protein [hsCRP] > 10 mg/L, leucocytosis [leukocyte count > 11 × 10^9^ cells/L], body temperature over 38°C, and those using antibiotics]) (*n* = 635), malignancy or history of malignancy (*n* = 734), patients who have a previous diagnosis of chronic liver disease or patients in chronic kidney disease classes G3b, G4, and G5 according to classification based upon estimated glomerular filtration rate (eGFR) < 45 mL/min (*n* = 603), and women receiving hormone replacement therapy (*n* = 39) were excluded from the analysis.

Patients' age, sex, body mass index (BMI), systolic blood pressure (SBP), smoking and alcohol consumption, biochemical results, including blood glucose, glycosylated hemoglobin, creatinine levels, and eGFR, LDL‐cholesterol, high‐density lipoprotein (HDL)‐cholesterol, triglycerides, total cholesterol, and Lp(a) levels were recorded. eGFR was calculated by using the Modification of Diet in Renal Disease (MDRD) formula [5]. Lp(a) was measured, using fresh plasma, with Roche Cobas Tina‐quant Lipoprotein(a) Gen 2 kit by immunoturbidimetric method at the certificated local laboratory. The lower limit of quantitation (LLoQ) for Lp(a) was 2 mg/dL. CRP levels were routinely measured using hsCRP assays with a calibrated kit from Roche. The cut‐off values for Lp(a) were determined as 30 and 50 mg/dL by considering the recommendations of the EAS consensus document and our data which indicated that the 75th percentile corresponds to an Lp(a) level of 29 mg/dL or higher [9].

Definitions for the clinical characteristics were used as follows. Hypertension was identified by a previous diagnosis or by office blood pressure measurements exceeding 140/90 mmHg on at least two occasions.

Diabetes was defined by a prior diagnosis, fasting blood glucose levels greater than 126 mg/dL, or HbA1c levels above 6.5%. Proteinuria was characterized by protein levels in spot urine exceeding 25 mg/dL, and CAD was defined by a history of acute coronary syndrome, documented stenosis of more than 50% in the epicardial coronary arteries (via conventional, computed tomography, or coronary angiography), evidence of myocardial ischemia on stress imaging, or a history of coronary revascularization. Due to the retrospective nature of the study, the initial CAD diagnosis of patients was made between 15 and 1 year ago when they were included in the study.

Statistical tests were conducted using the Statistical Package for the Social Sciences 26.0 for Windows (SPSS Inc., Chicago, IL, USA) and GraphPad Prism software version 9.4.1 (GraphPad Software, San Diego, USA). The normal distribution of the data was evaluated by the Kolmogorov–Smirnov test. Continuous data were expressed as mean ± standard deviation or median and 25th to 75th percentiles (Q1–Q3), and categorical data were expressed as percentages. Categorical variables were examined using the *χ* ^2^ test, while Student's *t* test and the Mann–Whitney *U* test were employed for normally distributed and non‐normally distributed continuous variables, respectively. Logistic regression analyses, both univariate and multivariate, were utilized to investigate the independent association of Lp(a) with CAD. To assess the predictability of Lp(a) level and Lp(a) cut‐off values ≥ 30 and ≥ 50 mg/dL for CAD, a multivariate regression model was constructed using the significant variables identified in univariate regression for CAD. Highly correlated variables such as SBP and hypertension or LDL cholesterol and total cholesterol were not used in the same model. Significance was assumed at a two‐sided *p* < 0.05.

## 3 Results

A total of 1858 patients with a mean age of 54.35 ± 17.05 years (53.3% female) with Lp(a) levels and fulfilling the inclusion–exclusion criteria were enrolled in the analysis. Hypertension was present in 37.5%, diabetes in 24.1%, and CAD in 26% of the cohort. The median Lp(a) level of the whole group was 12 mg/dL (Q1–Q3: 5–29 mg/dL), in men 11 mg/dL (Q1–Q3: 4–25 mg/dL) and in women 13 mg/dL (Q1–Q3: 6–32 mg/dL) (*p* < 0.001). The distribution of Lp(a) concentration in women and men is demonstrated in Supporting Information S1: Figures S1A and S1B, respectively. A total of 196 subjects had Lp(a) levels below the LLoQ, with no significant difference observed between men (*n* = 104, 8.3%) and women (*n* = 92, 8.4%) (*p* = 0.95). Upon evaluating 1858 patients' Lp(a) concentrations, it was observed that the 75th percentile corresponded to an Lp(a) level of 29 mg/dL or higher.

The basic characteristics of men and women in the study population are demonstrated in Supporting Information S1: Table S1. Men had higher percentages of smokers, hypertensives, and patients with proteinuria and CAD, as well as those on statins, beta blockers, and antiplatelet therapy, compared to women. Furthermore, men exhibited higher SBP and triglyceride levels, while women had higher eGFR and HDL‐cholesterol levels compared to men. Additionally, women had higher LDL and total cholesterol levels, which is probably associated with lower rates of statin usage. Lp(a) levels were observed to be higher in women (*p* < 0.001).

In the entire cohort, univariate analysis showed significant associations between CAD and the following variables, including age, sex, smoking habit, proteinuria, diabetes, hypertension, HDL‐cholesterol, LDL‐cholesterol, triglyceride levels, BMI, eGFR, and Lp(a) levels (Supporting Information S1: Table S2). The multivariate logistic regression model of these variables highlighted age, sex, diabetes, Lp(a) levels, and BMI as independent predictors of CAD.

**Figure 1:**
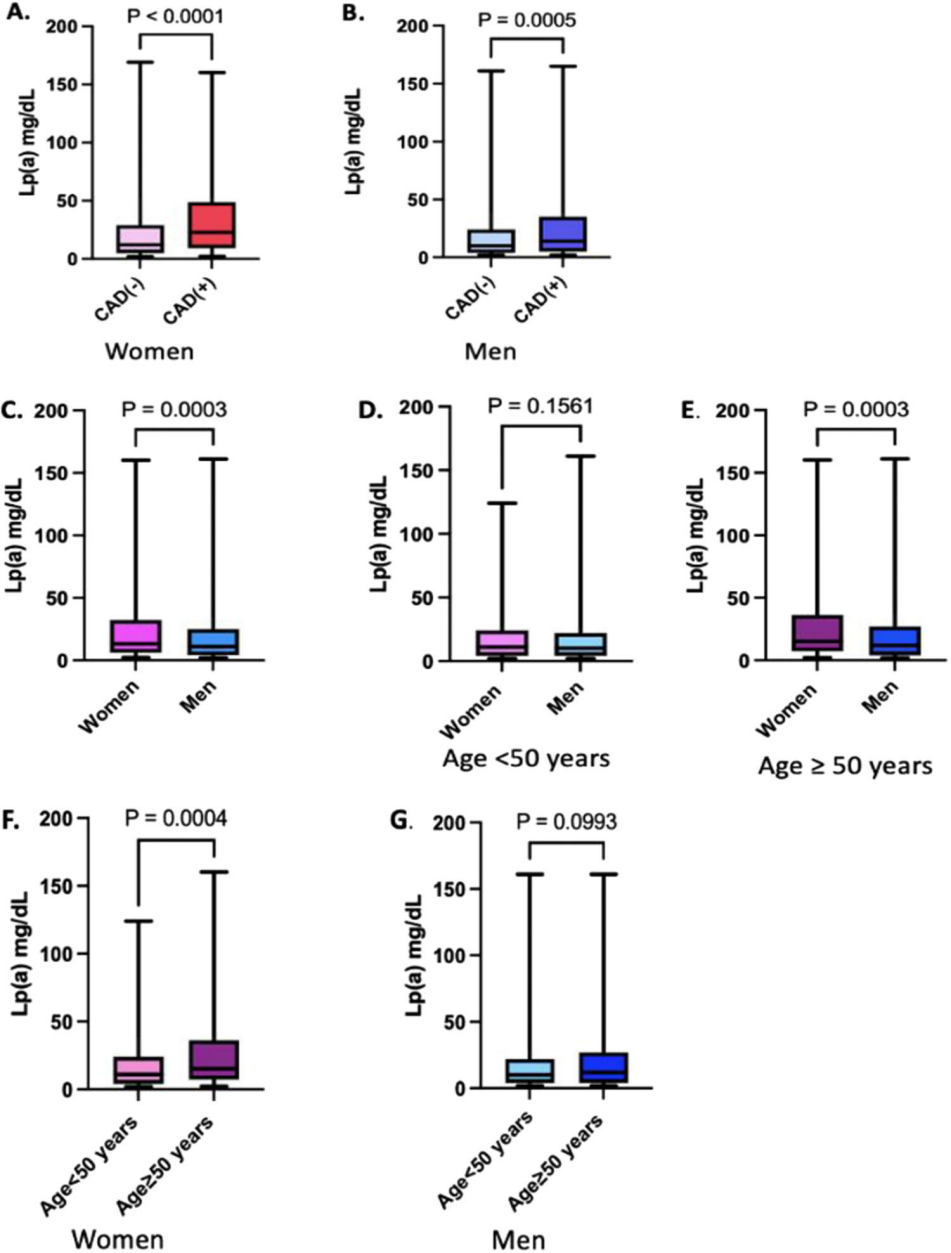
The distribution of Lp(a) concentration (mg/dL) in men and women. The box represents the interquartile range (IQR), the horizontal line inside the box indicates the median of the data, and the whiskers extend from the box to the smallest and largest values within 1.5 times the interquartile range (IQR). (A) Lp(a) concentration distribution in women according to the presence of CAD. (B) Lp(a) concentration distribution in men according to the presence of CAD. (C) Lp(a) concentration distribution in women and in men. (D) Lp(a) concentration distribution in women and men younger than 50 years of old. (E) Lp(a) concentration distribution in women and men 50 years old and older. (F) Lp(a) concentration distribution in women younger than 50 years, and in women 50 years and older. (G) Lp(a) concentration distribution in men younger than 50 years, and in men 50 years and older. CAD, coronary artery disease; Lp(a), lipoprotein(a).

Sex‐based analysis revealed that patients with CAD exhibited higher Lp(a) levels in both sexes (Figure 1A,B). Further analysis revealed that the rates of diabetes, hypertension, proteinuria, Lp(a) ≥ 50 mg/dL (*p* < 0.001), and Lp(a) ≥ 30 mg/dL (*p* = 0.05) were higher in the CAD group than in the non‐CAD group in men (Table 1). Likewise, women with CAD exhibited higher proportions of hypertension, diabetes, proteinuria, and Lp(a) ≥ 30 mg/dL (*p* < 0.001) as well as Lp(a) ≥ 50 mg/dL (*p* = 0.002). In both sexes, the proportion of those on statin therapy was higher among patients with CAD, leading to reduced total and LDL‐cholesterol levels in this group. The median HDL‐cholesterol level was lower, median triglyceride level was higher in CAD patients of both sexes compared to those without CAD.

**Table 1:** Characteristics of men and women according to the presence of coronary artery disease.

	Men (*n* = 978)			Women (*n* = 880)		
Variables	CAD (−) (*n* = 657)	CAD (+) (*n* = 321)	*p* value	CAD (−) (*n* = 773)	CAD (+) (*n* = 107)	*p* value
Demographic variables						
Age (mean ± SD)	47.1 ± 15.36	62.74 ± 12.17	< 0.001	50.32 ± 17.07	68.05 ± 11.09	< 0.001
BMI, m^2^/kg (mean ± SD)	26.97 ± 4.33	27.68 ± 4.02	0.021	26.63 ± 5.98	28.85 ± 5.86	< 0.001
Smokers, *n* (%)	223 (34.1)	110 (34.4)	0.364	190 (24.7)	24 (22.9)	0.389
Diabetes, *n* (%)	104 (15.8)	109 (34)	< 0.001	125 (16.2)	42 (39.3)	< 0.001
Hypertension, *n* (%)	182 (27.7)	177 (55.1)	< 0.001	213 (27.6)	66 (61.7)	< 0.001
SBP, mmHg (mean ± SD)	137.84 ± 45.1	125.05 ± 18.50	0.005	120.81 ± 14.03	127.17 ± 16.41	< 0.001
Age ≥ 50 years, *n* (%)	274 (41.8)	277 (85.8)	< 0.001	395 (51.1)	104 (97.2)	< 0.001
Medications						
Antiplatelets, *n* (%)	71 (10.8)	221 (68.7)	< 0.001	11 (9.4)	39 (63.6)	< 0.001
Beta blockers, *n* (%)	82 (12.5)	183 (57)	< 0.001	101 (13.1)	47 (53.3)	< 0.001
RAS blockers, *n* (%)	156 (16)	130 (40.5)	< 0.001	139 (18)	49 (45.8)	< 0.001
Statin users, *n* (%)	100 (10.2)	205 (63.9)	< 0.001	64 (8.3)	62 (57.9)	< 0.001
Biochemical analysis						
eGFR mL/min/1.73 m^2^ (mean ± SD)	99.93 ± 26.13	83.19 ± 26.08	< 0.001	101.2 ± 30.02	76.03 ± 27.78	< 0.001
Proteinuria, *n* (%)	95 (14.5)	72 (22.4)	0.020	78 (10.2)	25 (24.2)	0.003
Total cholesterol, mg/dL (mean ± SD)	203.40 ± 49.37	183.43 ± 52.62	< 0.001	212.72 ± 49.68	200.36 ± 55.55	0.009
HDL mg/dL (median, Q1–Q3) a	46 (39–55)	43 (37–50)	< 0.001	61 (51–72)	51.5 (43–64.5)	< 0.001
LDL, mg/dL (mean ± SD)	134.38 ± 42.36	116.13 ± 48.06	< 0.001	137.84 ± 45.10	125.05 ± 54.81	0.008
Trigliserid, mg/dL (median, Q1–Q3) a	131.5 (88.75–199)	150 (100–209)	0.018	103 (76–143.5)	134.5 (99.25–188.75)	< 0.001
hsCRP mg/L (mean ± SD)	1.88 ± 1.42	2.15 ± 1.42	0.013	1.98 ± 1.51	2.34 ± 1.44	0.041
Lp(a), mg/dL (median, Q1–Q3) a	10 (4–22)	13 (4.61–29.12)	< 0.001	12 (5–28.5)	22 (9–46)	< 0.001
Lp(a) ≥ 30 mg/dL, *n* (%)	128 (19.5)	78 (24.3)	0.050	184 (23.8)	48 (44.9)	< 0.001
Lp(a) ≥ 50 mg/dL, *n* (%)	53 (8.1)	45 (14)	0.003	94 (12.2)	25 (23.4)	0.002

Abbreviations: BMI, body mass index; CAD, coronary artery disease; eGFR, estimated glomerular filtration rate; HDL, high‐density lipoprotein; hsCRP, high sensitive CRP; LDL, low‐density lipoprotein; Lp(a), lipoprotein(a); Q1,25th percentile; Q3,75th percentile; SBP, systolic blood pressure.

^a^ Nonparametric test was used for statistical analysis.

Comparison based on age revealed that Lp(a) levels were significantly higher in women aged 50 years and older compared to those younger than 50 years, with median values of 15 mg/dL (Q1–Q3: 7–36 mg/dL) versus 11 mg/dL (Q1–Q3: 4–24 mg/dL), respectively (*p* = 0.016) (Figure 1F). Furthermore, the prevalence of CAD in women aged 50 years and over was 20.8%, a figure significantly higher than the 0.8% observed in those younger than 50 years old. Among women without CAD, 51% were 50 years of age or older; this proportion dramatically increased to 97.2% in women diagnosed with CAD (*p* < 0.001).

Although CAD prevalence was significantly higher in men aged 50 years and older compared to those younger than 50 years (50.3% vs. 10.8%, *p* < 0.001), no significant difference in Lp(a) levels was observed between the two age groups in men, with median values of 10 mg/dL (Q1–Q3: 4–22 mg/dL) for men aged 50 and over versus 11.9 mg/dL (Q1–Q3: 4–27 mg/dL) for those under 50 (*p* = 0.099) (Figure 1G). The proportion of patients with Lp(a) levels ≥ 30 mg/dL was similar across age groups in men, with 19.9% in those younger than 50 years and 21.1% in those 50 years and older (*p* = 0.690).

Although Lp(a) levels in women were generally higher than in men (Figure 1C), there was no significant difference between men and women under the age of 50 (*p* = 0.156) (Figure 1D). However, in individuals aged 50 years and older, women had significantly higher Lp(a) levels than men (*p* < 0.001) (Figure 1E).

**Figure 2:**
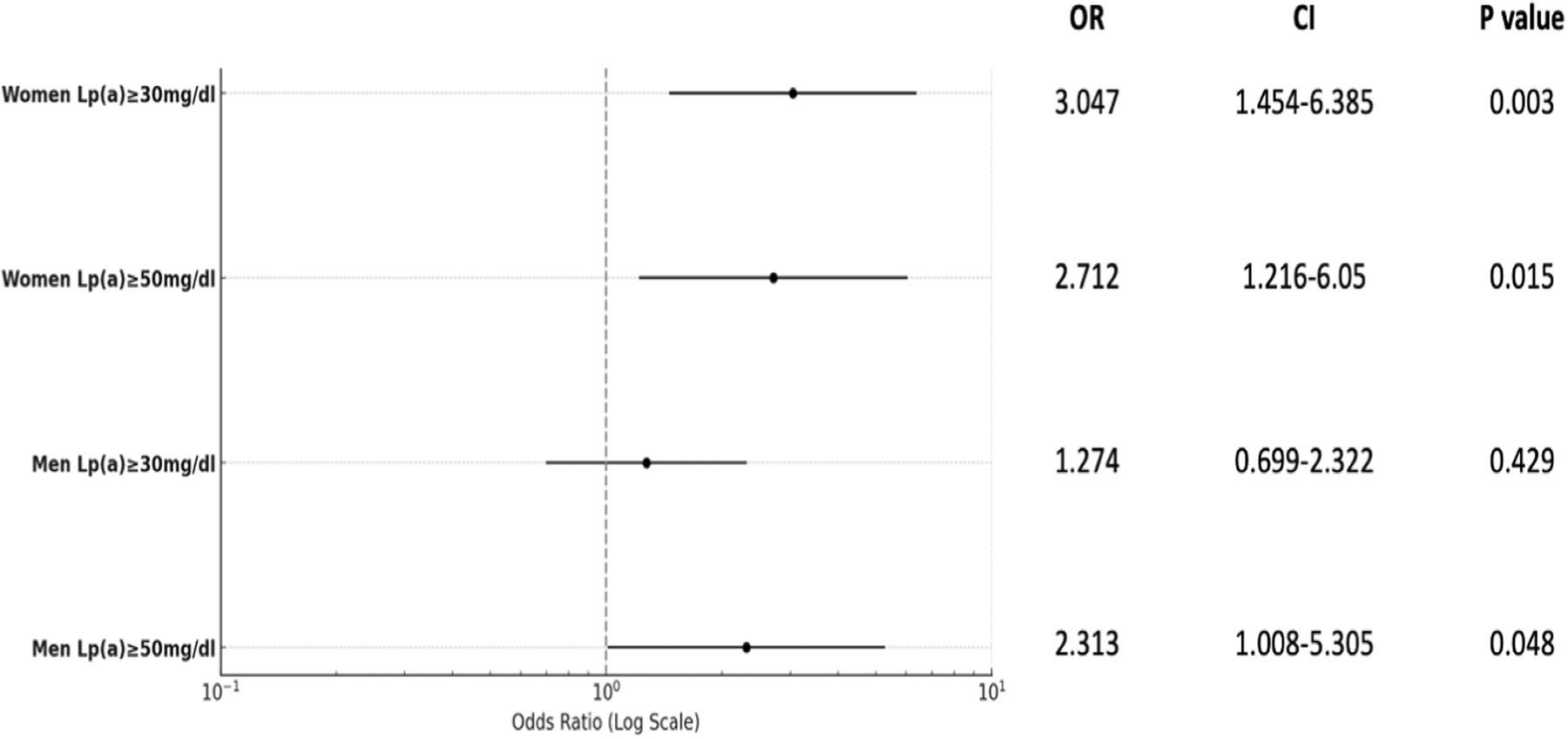
Odds ratios of different Lp(a) level cut‐offs in men and women for CAD prediction in multivariate analysis. CI, confidence interval; Lp(a), lipoprotein(a); OR, odds ratio.

In a multivariate logistic regression model, which was constructed using age, BMI, diabetes, hypertension eGFR, proteinuria, total cholesterol, HDL‐cholesterol, and Lp(a) level (log‐transformed Lp(a) was used in the model due to skewed distribution), the Lp(a) level was found to be an independent predictor of CAD in both men and women (men: OR: 1.36, 95% CI: 1.030–1.810, *p* = 0.03; women: OR: 2.0206, 95% CI: 1.464–3.324, *p* < 0.001, per 10‐fold Lp(a) level increment). When incorporating Lp(a) level cut‐off values of ≥ 30 mg/dL and ≥ 50 mg/dL into the model successively, it was demonstrated that an Lp(a) level of ≥ 30 mg/dL predicted CAD exclusively in women, with no similar effect observed in men. Conversely, an Lp(a) level of ≥ 50 mg/dL independently predicted CAD across both sexes, as shown in Table 2 and Figure 2.

**Table 2:** Independent predictors of CAD in men and women.

	Multivariate analysis			
	OR	OR corresponds to increment	95% CI	*p* value
Men				
Age	1.082	Per year	1.053–1.111	**< 0.001**
BMI	1.105	Per 1 kg/m^2^	1.035–1.179	**0.003**
Diabetes	1.454	Per 1 kg/m^2^	0.846–2.498	0.175
Hypertension	1.016	Diabetes versus Without Diabetes	0.594–1.738	0.953
eGFR	1.002	Per 1 mL/min/1.73 m^2^	0.990–1.014	0.776
Proteinuria	1.309	Proteinuria versus without proteinuria	0.715–2.397	0.382
Total cholesterol	1	Per 1 mg/dL	0.996–1.005	0.959
HDL	0.967	Per 1 mg/dL	0.945–0.990	**0.005**
CRP	0.893	Per 1 g/L	0.735–1.086	0.258
Lp(a) ≥ 30 mg/d^a^	1.274	Lp(a) ≥ 30 mg/dL versus Lpa < 30 mg/dL	0.699–2.322	0.429
Lp(a) ≥ 50 mg/dL^a^	2.313	Lp(a) ≥ 50 mg/dL versus Lp(a) < 50 mg/dL	1.008–5.305	**0.048**
Women				
Age	1.053	Per year	1.019–1.089	**0.002**
BMI	1.052	Per 1 kg/m^2^	0.987–1 1.122	0.119
Diabetes	2.132	Per 1 kg/m^2^	1.014–4.482	**0.046**
Hypertension	1.543	Diabetes versus without diabetes	0.678–3.507	0.301
eGFR	0.997	Per 1 mL/min/1.73 m^2^	0.983–1.011	0.682
Proteinuria	1.039	Proteinuria versus without proteinuria	0.406–2.659	0.936
Total cholesterol	0.095	Per 1 mg/dL	0.985–1.001	0.180
HDL	0.975	Per 1 mg/dL	0.95–1	0.054
CRP	1.011	Per 1 g/L	0.795–1.285	0.93
Lp(a) ≥ 30 mg/dL^a^	3.047	Lp(a) ≥ 30 mg/dL versus Lp(a) < 30 mg/dL	1.454–6.385	**0.003**
Lp(a) ≥ 50 mg/dL^a^	2.712	Lp(a) ≥ 50 mg/dL versus Lp(a) < 50 mg/dL	1.216–6.05	**0.015**

*Note:* Bold values are statistically significant.

Abbreviations: BMI, body mass index; CAD, coronary artery disease; eGFR, estimated glomerular filtration rate; HDL, high‐density lipoprotein; LDL, low‐density lipoprotein; Lp(a), lipoprotein (a); SBP, systolic blood pressure.

^a^ Lp(a) ≥ 30 mg/dL and Lp(a) ≥ 50 mg/dL were included in the multivariate analysis model separately in each group.

## 4 Discussion

Our study provided valuable insights into Lp(a) levels among a large Turkish cohort. In the study group, the median Lp(a) level was 12 mg/dL, and Lp(a) was an independent predictor of CAD for the entire cohort. Women exhibited higher Lp(a) levels than men. Despite Lp(a) level being higher in women, Lp(a) cut‐off level of ≥ 30 mg/dL was a predictor of CAD only in women; however, both men and women with Lp(a) levels of ≥ 50 mg/dL were found to be at increased risk for CAD.

Elevated levels of Lp(a) are widely recognized as a significant risk factor for CAD. Previous large‐scale population studies have demonstrated that Lp(a) levels are generally higher in women than in men, raising questions about potential sex differences in the Lp(a)‐CAD relationship [18, 19]. Our findings indicate that elevated Lp(a) levels are associated with CAD in both sexes, suggesting that the risk posed by high Lp(a) levels transcends sex differences. Specifically, in our cohort, patients with CAD, irrespective of being male or female, exhibited higher Lp(a) levels. This observation may underscore the importance of considering elevated Lp(a) levels as a critical risk factor for CAD across both sexes.

While the association between Lp(a) levels and CAD is well‐established, the specific threshold of Lp(a) that accurately predicts CAD risk remains ambiguous. A significant meta‐analysis demonstrated that an Lp(a) level exceeding 30 mg/dL is associated with an increased risk of coronary heart disease, irrespective of sex differences [20]. Current guidelines, however, propose varying Lp(a) thresholds for identifying high‐risk individuals. The ACC/AHA, CCS, and the National Lipid Association (NLA) all recognize 50 mg/dL (or > 100 nmol/L) as a risk‐enhancing cut‐off [7, 8]. Conversely, the EAS consensus statement delineates < 30 mg/dL (or < 75 nmol/L) as normal, 30–50 mg/dL (or 75–125 nmol/L) as intermediate, and ≥ 50 mg/dL (or > 125 nmol/L) as indicative of an abnormal level [9].

Discrepancies in recommendations regarding Lp(a) level cut‐offs can be attributed to several factors. Firstly, despite efforts to standardize kits and measurement methods, a global standardization of Lp(a) measurement techniques has yet to be achieved. This lack of uniformity complicates comparative analyses across different regions. Secondly, it is well‐documented that average Lp(a) levels vary significantly among different populations and ethnic groups [12, 21]. For instance, a study from the UK Biobank reported a median Lp(a) level of 19 nmol/L (equivalent to approximately 7.6 mg/dL based on the EAS guidelines in a predominantly White population [18]. In contrast, our study, which also focused on a White demographic, found a slightly higher median Lp(a) level of 12 mg/dL, surpassing the levels typically observed in European cohorts. This finding aligns with prior research conducted within the Turkish population, which identified average Lp(a) levels ranging from 11 mg/dL to 15 mg/dL in the general adult population [22]. These observations underscore the influence of ethnic and regional variations on Lp(a) levels, further complicating the establishment of a universal cut‐off threshold.

In large general population studies, Lp(a) levels have been consistently observed to be higher in women than in men, a finding that aligns with our findings. In our study, the Lp(a) level was higher in women than in men. Furthermore, even though statin treatment, which is known to be related to Lp(a) level increment, was more frequent in men, women had higher Lp(a) levels than men, similar to Odyssey Study findings [23, 24].

Despite the difference in Lp(a) levels in men and women, current guidelines do not offer distinct Lp(a) level thresholds for men and women. However, observed sex‐specific variations in Lp(a) levels may hint at the potential need for sex‐specific thresholds to more accurately predict CAD. Numerous studies have highlighted that the association between Lp(a) levels and cardiovascular risk is influenced by sex. Nonetheless, research focusing on these sex differences has yielded inconsistent findings.

The Framingham Offspring Study identified a significant relationship between Lp(a) levels and CVD risk only in men [25]. Another study suggested that the relevance of Lp(a) levels to vascular mortality is confined to older men, with no significant association found in older women [26]. Cook et al. concluded from their analysis of three large cohorts that an increased Lp(a) level signifies a heightened risk for CVD in women, but only when total cholesterol levels exceed 220 mg/dL [27]. Similarly, Bigazzi et al. found that high Lp(a) levels were associated with increased revascularisation needs in women with CAD, a correlation not observed in men [15]. Major studies that analyzed Lp(a) level and its association with CVD in men and women are summarized in Table 3. Despite the disparate findings, the results of these studies suggest that the predictive cut‐off value of Lp(a) level for CAD may differ between sexes. In our analysis, while elevated Lp(a) levels and a prevalence of Lp(a) ≥ 30 mg/dL were observed in CAD patients of both sexes, Lp(a) ≥ 30 mg/dL was an independent predictor of CAD only in women. In men, an Lp(a) level of ≥ 30 mg/dL was not a predictor for CAD; however, an Lp(a) level of ≥ 50 mg/dL was indicative of CAD risk. These observations may imply that women, despite having higher median Lp(a) levels, could be more susceptible to the atherosclerotic effects of Lp(a), developing CAD at lower Lp(a) concentration.

**Table 3:** Studies that compare Lp(a) levels its association with CVD in men and women.

Reference	Study design and study group	Median Lp(a) level in men OR % of men who had defined Lp(a) levels		Median Lp(a) level in women OR % of women who had defined Lp(a) levels		Cut‐off level used for analysis	End point	Hazard/odds ratio in men	Hazard/odds ratio in women
Ariyo et al. [26]	3972 subjects from Cardiovascular Health Study fallowed for a median 7.4 years	3.9 mg/dL		4.4 mg/dL		Patients were divided into five groups according to Lp(a) level quintiles. HR analyzed by assuming HR for the lowest quintile is 1. In this table, HRs for the highest quintile were given	Stroke	2.92 (95% CI: 1.53–5.57)	1.11 (95% CI: 0.70–1.78)
							Death from vascular causes	2.09 (95% CI: 1.27–3.47)	0.87 (95% CI: 0.52–1.40)
							Death from all causes	0.96 (95% CI: 0.67–1.30)	1.60 (95% CI: 1.16–2.19)
							CAD	1.02 (95% CI: 0.71–1.46)	1.19 (95% CI: 0.84–1.67)
Lamon‐Fava et al. [25]	1328 men and 1562 women from Framingham Offspring Study who do not have CAD and had their plasma Lp(a) levels measured	CAD (−)	CAD (+)	CAD (−)	CAD (+)	—	CAD	2.57 (95% CI: 1.5–1.4.4)	Was not calculated due to nonsignificant Lp(a) level difference between women with CAD and without CAD
		10.8 mg/dL	20.6 mg/dL	13 mg/dL	12 mg/dL				
Patel et al. [18]	460 506 participants in whom Lp(a) concentrations were measured from UK biobank cohort	17.4 nmol/L		21.77 nmol/L		Without cut‐off	Risk of atherosclerotic cardiovascular disease and ischemic stroke	1.11 (95% CI: 1.10–1.12)	1.10 (95% CI: 1.09–1.11)
Bigazzi et al. [15]	2374 patients admitted to coronary care unit with ischemia were enrolled	Patients with Lp(a) ≤ 30 mg/dL (%)	66.74%	65.5%		Lp(a) ≤ 30 mg/dL	Major cardiovascular events	1	1
		Patients with 30 mg/dL < Lp(a) < 50 mg/dL (%)	15.3%	14.19%		Lp(a) > 30–50 mg/dL		1.24 (95% CI: 0.81–1.90)	1.91 (95% CI: 0.65–5.55)
		Patients with Lp(a) ≥ 50 mg/dL (%)	17.94%	20.21%		Lp(a) ≥ 50 mg/dL		1.22 (95% CI: 0.82–1.81)	4.15 (95% CI: 1.85–9.25)
Simony et al. [19]	37 545 Danish women, 32 497 Danish men from general population	29.065 mg/dL		33.770 mg/dL		< 10 mg/dL versus > 40 mg/dL	Myocardial infection	1.56 (95% CI: 1.38–1.77)	1.44 (95% CI: 1.23–1.69)
Pino et al. [28]	2110 coronary angiography patients who are older than 50 years old and followed up for 10 years	Patients with Lp(a)> 30 mg/dL (%)	45%	49%		Lp(a) > 30 mg/dL	Hard events include mortality and nonfatal myocardial infarction	Data were not given	With type 2 diabetes: 2.9 (95% CI: 1.1–7.7)
Yurtseven et al. (This study)	1858 patients admitted to cardiology and internal medicine outpatient clinics	11 mg/dL		13 mg/dL		Lp(a) ≥ 30 mg/dL	CAD	0.42 (95% CI: 0.69–2.32)	0.003 (95% CI: 1.45−6.38)
						Lp(a) ≥ 50 mg/dL		0.048 (95% CI: 1.0–5.30)	0.015 (95% CI: 1.21–6.05)

Abbreviations: CAD, coronary artery disease; CI, confidence interval; HR, hazard ratio; Lp(a) lipoprotein a.

Given Lp(a)'s strong association with inflammation, we propose that the increased susceptibility of women to Lp(a) might be due to the higher levels of inflammation observed in women. The accumulation of Lp(a) within arterial walls elicits an inflammatory response, marked by increased secretion of chemoattractants and cytokines. Moreover, the *LPA* gene features an IL6 response element, implicating it in systemic inflammatory responses, such as sepsis, which consequently elevates Lp(a) levels [17]. Consistently, ACCELERATE trial demonstrated that in CAD patients with higher hsCRP levels (hsCRP > 2 mg/L) and Lp(a) > 30 mg/dL had an increased risk of recurrent events compared to those with hsCRP < 2 mg/L and Lp(a) > 30 mg/dL [29]. However, the Copenhagen Population Study found no correlation between hsCRP and Lp(a) levels. Moreover, when the data were analyzed for men and women, the ability of Lp(a) levels to predict CVD risk was not influenced by hsCRP levels in either sex [30]. In our study, the increased risk associated with Lp(a) levels exceeding 30 mg/dL was observed exclusively in women, which we hypothesized might be linked to inflammation. However, no significant differences in hsCRP levels were detected between men and women. Although this finding is consistent with the analysis of the Copenhagen Study, it should be considered that this may be related to the exclusion of patients with inflammation or selection bias.

Diabetes has been identified as a factor influencing Lp(a) metabolism, with previous research suggesting that diabetes is associated with lower levels of Lp(a) [31]. In our study, the prevalence of diabetes was similar in men and women, indicating that the observed lower Lp(a) levels in males cannot be attributed to differences in diabetes prevalence. Pino et al. found Lp(a) as a predictor of CVD only in women with type 2 diabetes, but a relationship was not observed in men or women without diabetes [28]. In our study in both men and women, diabetes was more frequent in patients with CAD, but diabetes was an independent predictor of CAD only in women when analyzed in the multivariable regression model. The susceptibility of women to lower levels of Lp(a) may be related to the additive CAD risk increment of diabetes in women.

Copenhagen General Population Study has highlighted a significant rise in Lp(a) levels in women post‐50 years of age, suggesting that elevated Lp(a) levels constitute a more prevalent risk factor for CVD in women above 50 years of age [17]. This observation aligns with our findings, where 97.4% of women diagnosed with CAD and 70.9% of those with Lp(a) levels equal to or exceeding 30 mg/dL were aged 50 years or older. Additionally, while Lp(a) levels before 50 years of age were comparable between men and women, a marked increase was observed exclusively in women beyond this age threshold, coinciding with an overall rise in CAD prevalence in both sexes. This trend may indicate a more pronounced correlation between Lp(a) levels and CAD risk among women over 50 years old. The rise in the Lp(a) level around 50 years of age could be due to hormonal changes related to menopause. The regulatory influence of estrogen on the *LPA* gene is well‐documented, and numerous studies corroborate a postmenopausal surge in Lp(a) levels in women [17]. Similarly, bilateral oophorectomy has been linked to elevated Lp(a) levels [26]. Such increases in Lp(a) levels in postmenopause may contribute significantly to the escalated CVD risk observed in this group [27]. Furthermore, the ESC Women, Lipids, and Atherosclerotic CVD Call to Action paper suggests that a once‐in‐a‐lifetime measurement of Lp(a) may not be sufficient for women [32].

Our study has several limitations that warrant consideration. It is retrospective nature, which may introduce selection bias and limit the ability to establish causality. Additionally, the heterogeneity of the study population and single‐center cross‐sectional nature could affect the generalizability of the findings. Information regarding menopausal status was not collected in our study; therefore, we assumed a threshold age of 50 years as a proxy for menopause in our analyses. Moreover, Lp(a) levels were measured in mass concentration units, as opposed to the molar concentration units recently recommended by clinical guidelines. Despite this, the mass concentration method remains widely used in clinical practice, which may aid in comparing our results with existing literature.

Among the strengths of our research is the considerable sample size drawn from a single center, which enhances the internal consistency of our findings. The standardization of Lp(a) measurements, conducted in a controlled environment using fresh plasma samples at the time of patient admission, further supports the reliability of our data. The analyses were carried out in a certified university hospital laboratory, with routine quality control procedures, including calibration of the analytical equipment. Additionally, our study contributes to the limited body of research exploring the influence of sex on the Lp(a)‐CAD relationship, thereby providing valuable insights into this clinically important interplay.

## 5 Conclusion

Our study's findings within a Turkish cohort reveal that elevated Lp(a) levels are associated with an increased risk of CAD in both men and women. Notably, while an Lp(a) level of ≥ 50 mg/dL was predictive of CAD across both sexes, Lp(a) level of ≥ 30 mg/dL emerged as an independent predictor exclusively in women. These results may underscore the potential for sex‐specific thresholds in the clinical evaluation of CAD risk related to Lp(a) levels. Future research, designed as prospective studies and encompassing larger cohorts, will be essential to substantiate our findings and may further delineate a possible sex‐dependent predictivity of Lp(a) levels for CAD.

## Author Contributions

Ece Yurtseven analyzed the data and wrote the article. Dilek Ural designed the study, directed the research, and edited the article. Erol Gursoy collected data and contributed to data analysis. Bekay Omer Cunedioglu collected data. Orhan Ulas Guler collected data. Kemal Baysal contributed to biochemical analysis, data collection, and data analysis. Saide Aytekin contributed to data collection and writing of the article. Vedat Aytekin contributed to data collection and data analysis. Meral Kayikcioglu contributed to the writing of the article, designed the study, directed the research, and reviewed and edited the article.

## Conflicts of Interest

Meral Kayikcioglu has received honoraria from Abbott, Abdi Ibrahim, Chiesi, LIB Therapeutics, Novartis, Novo Nordisk, TR‐pharma, and Ultragenix; research funding from Amryt Pharma, and has participated in clinical trials with Amgen, Ionis, LIB Therapeutics, Novartis, Novo Nordisk, during the past 3 years. Dilek Ural has received honoraria from Amgen, Astra Zeneca, Boehringer Ingelheim, and Novartis; has participated in clinical trials with Vifor International during the past 3 years. Vedat Aytekin has received honoraria from Chiesi, Sanofi, Pfizer, and Recordati during the past 3 years. The other authors declare no conflicts of interest.

## Supporting information

Supporting information.

Supporting information.

## Data Availability

Data used in this study are available upon reasonable request to the corresponding author, subject to the approval of all the authors.
