# Supplementary material for: Is There a Need for Sex‐Tailored Lipoprotein(a) Cut‐Off Values for Coronary Artery Disease Risk Stratification?
Source: Clin Cardiol. 2024 Sep 12;47(9):e70012. doi: 10.1002/clc.70012 (PMC11391382; doi:10.1002/clc.70012)
Supplement: Supplementary file 1 — Supporting information. [file CLC-47-e70012-s001.docx]

**Supporting Information Table S1.** Characteristics of Study Group

|  | Men  (n=978) | Women  (n=880) | p value |
| --- | --- | --- | --- |
| *Demographic variables* | | | |
| Age (mean± SD) | 52.3±16.16 | 52.47±17.45 | 0.759 |
| BMI,m^2^/ kg (mean± SD) | 27.2±4.23 | 26.95±6.01 | 0.280 |
| Smokers n (%) | 337 (34.5) | 215 (24.5) | <0.001 |
| Diabetes n (%) | 213 (21.8) | 177 (19) | 0.150 |
| Hypertension n (%) | 359 (36.7) | 279 (31.7) | 0.024 |
| CAD n (%) | 321 (32.8) | 1107 (2.2) | <0.001 |
| SBP,mmHg (mean± SD) | 123.79±15.4 | 121.6±14.49 | <0.001 |
| *Medications* | | | |
| Antiplatelets n (%) | 293 (30) | 141 (16) | <0.001 |
| Beta-blockers n (%) | 265 (27.1) | 158 (18) | <0.001 |
| RAS blockers n (%) | 235 (24) | 188 (21.4) | 0.184 |
| Statins n (%) | 272 (27.8) | 126 (14.3) | <0.001 |
| *Biochemical analysis* | | | |
| eGFR ml/min/1.73m^2^ (mean± SD) | 94.29±27.27 | 97.91±30.91 | 0.01 |
| Proteinuria n (%) | 168 (17.2) | 107 (12.2) | 0.041 |
| Total cholesterol, mg/dL(mean± SD) | 196.83±51.30 | 211.21±50.56 | <0.001 |
| HDL mg/dL(median, Q1-Q3)* | 45 (38-53.7) | 60 (50-71) | 0.000 |
| LDL, mg/dL(mean± SD) | 128.37±45.12 | 136.28± 45.54 | <0.001 |
| Triglycerides, mg/dL(median, Q1-Q3)* | 136(92-199) | 105 (78-149) | 0.000 |
| hsCRP mg/L (mean± SD) | 1.97±1.42 | 2.04±1.51 | 0.421 |
| Lp(a), mg/dL(median, Q1-Q3)* | 11 (4-25) | 13 (6-32) | <0.001 |
| Lp(a) ≥30 mg/dL n (%) | 201 (20.6) | 225 (25.6) | 0.011 |
| Lp(a) ≥50 mg/dL n (%) | 99 (10.1) | 119 (13.5) | 0.025 |
| BMI, body mass index; CAD, coronary artery disease; eGFR, estimated glomerular filtration rate; HDL, high density lipoprotein; hsCRP, high sensitive CRP; LDL,low density lipoprotein; Lp(a), lipoprotein (a); Q1,25th percentile; Q3, 75th percentile; SBP, systolic blood pressure  **Nonparametric test was used for statistical analysis* | | | |

**Supporting Information Table S2.** Independent Associates of CAD for the Study population

| Variables | OR | OR Corresponds to Increment | 95%CI | p value |
| --- | --- | --- | --- | --- |
| Sex (female) | 0.230 | Female vs Male | 0.16-0.333 | **<0.001** |
| Age | 1.058 | Per year | 1.043-1.073 | **<0.001** |
| BMI | 1.058 | Per 1 kg/m^2^ | 1.026-1.091 | **<0.001** |
| Smoking | 1.26 | Smoking vs Nonsmoking | 0.889-1.782 | 0.129 |
| Hypertension | 1.078 | Hypertension vs Without hypertension | 0.773-1.503 | 0.659 |
| Diabetes | 1.69 | Diabetes vs Without Diabetes | 1.222-2.345 | **0.001** |
| eGFR | 1.001 | Per 1 ml/min/1.73m^2^ | 0.995-1.007 | 0.830 |
| Proteinuria | 1.045 | Proteinuria vs Without Proteinuria | 0.73-1.511 | 0.815 |
| LDL-cholesterol | 1.001 | Per 1mg/dL | 0.994-0.997 | 0.094 |
| Triglyceride | 1.000 | Per 1mg/dL | 0.999-1.000 | 0.422 |
| HDL-cholesterol | 0.987 | Per 1mg/dL | 0.975-0.999 | **0.030** |
| **Lp(a)*** | 1.012 | Per log transformed unit (per10 fold increase) | 1.007-1.018 | **<0.001** |
| BMI, body mass index; CAD,coronary artery disease; eGFR, estimated glomerular filtration rate; HDL, high density lipoprotein; LDL,low density lipoprotein; Lp(a), lipoprotein (a) * *Due to skewed distribution, log transformed values were used in the analysis*. | | | | |


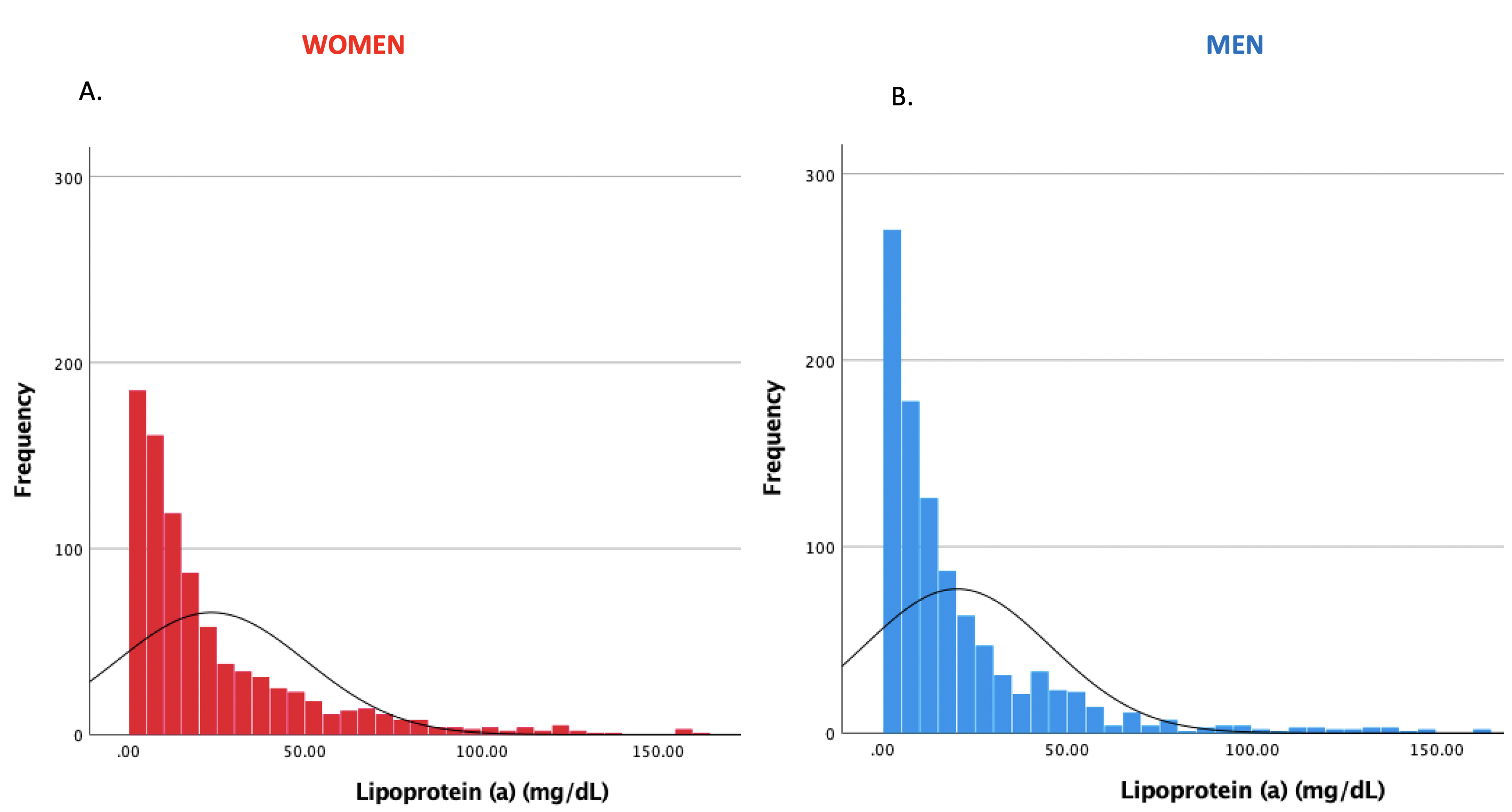


**Supporting Information Figure S1**. Frequency distribution of Lp(a) concentration (mg/dL) in men and women. **A**. Frequency distribution of Lp(a) concentration (mg/dL) in women. **B**. Frequency distribution of Lp(a) concentration (mg/dL) in men. Lp(a), lipoprotein (a)
