# Supplementary material for: Is There a Need for Sex‐Tailored Lipoprotein(a) Cut‐Off Values for Coronary Artery Disease Risk Stratification?
Source: Clin Cardiol. 2024 Sep 12;47(9):e70012. doi: 10.1002/clc.70012 (PMC11391382; doi:10.1002/clc.70012)
Supplement: Supplementary file 2 — Supporting information. [file CLC-47-e70012-s002.docx]

**Supporting Information For CLC-24-0425.R1; Is There a Need for Sex-Tailored Lipoprotein (a) Cut-Off Values for Coronary Artery Disease Risk Stratification?**

1. Dr. Ece Yurtseven, MD, PhD: Koc University School of Medicine, Department of Cardiology
2. Prof. Dr. Dilek Ural, MD: Koc University School of Medicine, Department of Cardiology
3. Assoc. Prof. Erol Gursoy, MD : Koc University School of Medicine, Department of Cardiology
4. Dr. Bekay Omer Cunedioglu, MD: Koc University School of Medicine
5. Dr. Orhan Ulas Guler, MD : Koc University School of Medicine
6. Prof. Dr. Kemal Baysal, MD, PhD: Koc University School of Medicine, Department of Biochemistry
7. Prof. Dr. Saide Aytekin: Koc University School of Medicine, Department of Cardiology
8. Prof. Dr. Vedat Aytekin: Koc University School of Medicine, Department of Cardiology
9. Prof. Dr. Meral Kayakcioglu: Ege University School of Medicine, Department of Cardiology

Corresponding Author: Ece Yurtseven, MD, PhD

Email address of the corresponding author: [eyurtseven@ku.edu.tr](mailto:eyurtseven@ku.edu.tr)

Telephone number of the corresponding author: mobile: +90 5326685490

Work: +90 850 250 8 250/ 29781

Content: We have included two tables and one figure related to the data presented in the article. Due to the limitations on the number of figures and tables allowed in the revised manuscript, as communicated to us during submission, we have provided these tables as supporting information

**Supporting Information Table S1.** Characteristics of Study Group

|  | Men  (n=978) | Women  (n=880) | p value |
| --- | --- | --- | --- |
| *Demographic variables* | | | |
| Age (mean± SD) | 52.3±16.16 | 52.47±17.45 | 0.759 |
| BMI,m^2^/ kg (mean± SD) | 27.2±4.23 | 26.95±6.01 | 0.280 |
| Smokers n (%) | 337 (34.5) | 215 (24.5) | <0.001 |
| Diabetes n (%) | 213 (21.8) | 177 (19) | 0.150 |
| Hypertension n (%) | 359 (36.7) | 279 (31.7) | 0.024 |
| CAD n (%) | 321 (32.8) | 1107 (2.2) | <0.001 |
| SBP,mmHg (mean± SD) | 123.79±15.4 | 121.6±14.49 | <0.001 |
| *Medications* | | | |
| Antiplatelets n (%) | 293 (30) | 141 (16) | <0.001 |
| Beta-blockers n (%) | 265 (27.1) | 158 (18) | <0.001 |
| RAS blockers n (%) | 235 (24) | 188 (21.4) | 0.184 |
| Statins n (%) | 272 (27.8) | 126 (14.3) | <0.001 |
| *Biochemical analysis* | | | |
| eGFR ml/min/1.73m^2^ (mean± SD) | 94.29±27.27 | 97.91±30.91 | 0.01 |
| Proteinuria n (%) | 168 (17.2) | 107 (12.2) | 0.041 |
| Total cholesterol, mg/dL(mean± SD) | 196.83±51.30 | 211.21±50.56 | <0.001 |
| HDL mg/dL(median, Q1-Q3) | 45 (38-53.7) | 60 (50-71) | 0.000 |
| LDL, mg/dL(mean± SD) | 128.37±45.12 | 136.28± 45.54 | <0.001 |
| Triglycerides, mg/dL(median, Q1-Q3) | 136(92-199) | 105 (78-149) | 0.000 |
| hsCRP mg/L (mean± SD) | 1.97±1.42 | 2.04±1.51 | 0.421 |
| Lp(a), mg/dL(median, Q1-Q3) | 11 (4-25) | 13 (6-32) | <0.001 |
| Lp(a) ≥30 mg/dL n (%) | 201 (20.6) | 225 (25.6) | 0.011 |
| Lp(a) ≥50 mg/dL n (%) | 99 (10.1) | 119 (13.5) | 0.025 |
| BMI, body mass index; CAD, coronary artery disease; eGFR, estimated glomerular filtration rate; HDL, high density lipoprotein; hsCRP, high sensitive CRP; LDL,low density lipoprotein; Lp(a), lipoprotein (a); Q1,25th percentile; Q3, 75th percentile; SBP, systolic blood pressure  **Nonparametric test was used for statistical analysis* | | | |

**Supporting Information Table S2.** Independent Associates of CAD for the Study population

| Variables | OR | OR Corresponds to Increment | 95%CI | p value |
| --- | --- | --- | --- | --- |
| Sex (female) | 0.230 | Female vs Male | 0.16-0.333 | **<0.001** |
| Age | 1.058 | Per year | 1.043-1.073 | **<0.001** |
| BMI | 1.058 | Per 1 kg/m^2^ | 1.026-1.091 | **<0.001** |
| Smoking | 1.26 | Smoking vs Nonsmoking | 0.889-1.782 | 0.129 |
| Hypertension | 1.078 | Hypertension vs Without hypertension | 0.773-1.503 | 0.659 |
| Diabetes | 1.69 | Diabetes vs Without Diabetes | 1.222-2.345 | **0.001** |
| eGFR | 1.001 | Per 1 ml/min/1.73m^2^ | 0.995-1.007 | 0.830 |
| Proteinuria | 1.045 | Proteinuria vs Without Proteinuria | 0.73-1.511 | 0.815 |
| LDL-cholesterol | 1.001 | Per 1mg/dL | 0.994-0.997 | 0.094 |
| Triglyceride | 1.000 | Per 1mg/dL | 0.999-1.000 | 0.422 |
| HDL-cholesterol | 0.987 | Per 1mg/dL | 0.975-0.999 | **0.030** |
| **Lp(a)*** | 1.012 | Per log transformed unit (per10 fold increase) | 1.007-1.018 | **<0.001** |
| BMI, body mass index; CAD,coronary artery disease; eGFR, estimated glomerular filtration rate; HDL, high density lipoprotein; LDL,low density lipoprotein; Lp(a), lipoprotein (a) * *Due to skewed distribution, log transformed values were used in the analysis*. | | | | |


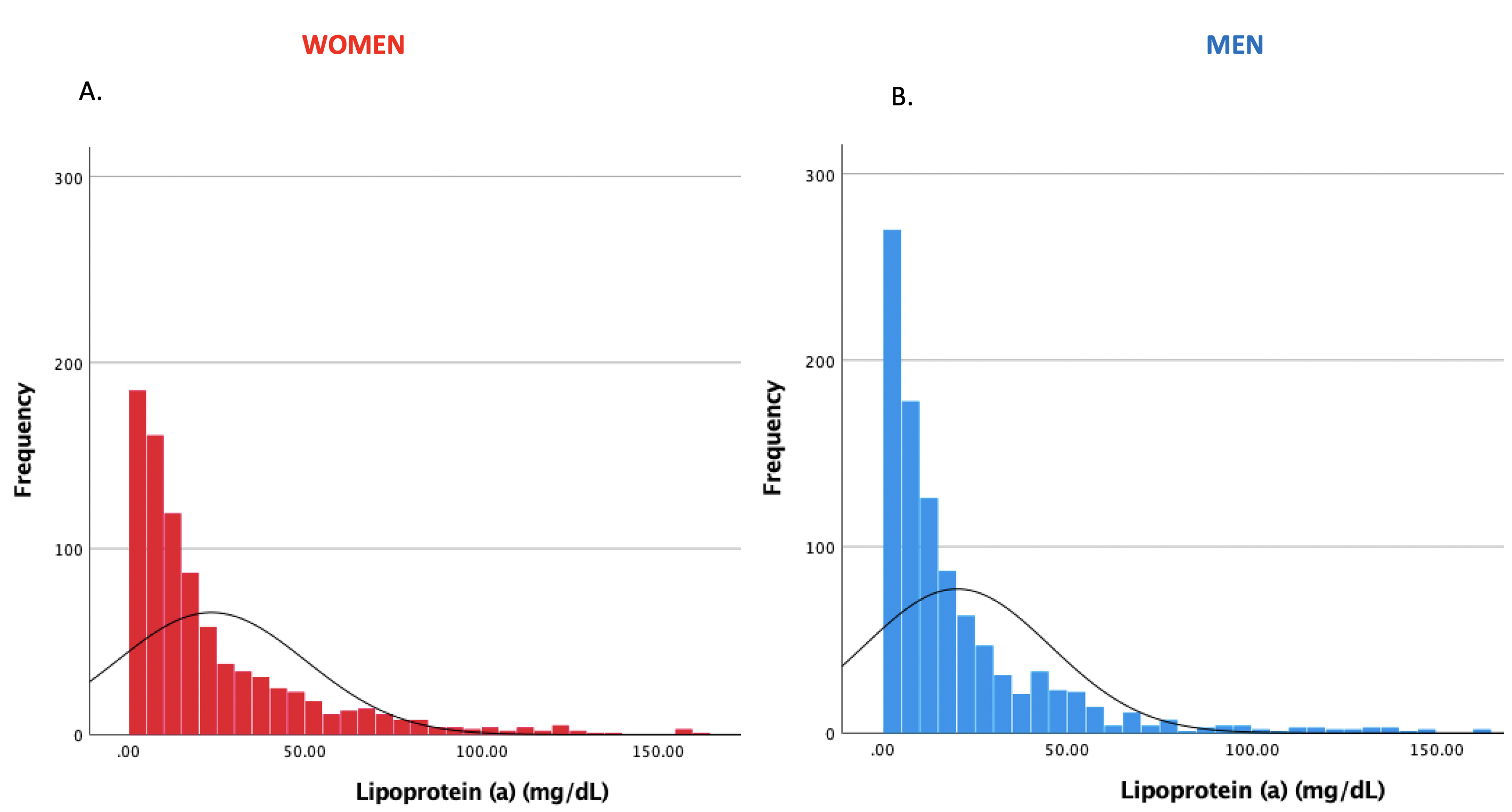


**Supporting Information Figure S1**. Frequency distribution of Lp(a) concentration (mg/dL) in men and women. **A**. Frequency distribution of Lp(a) concentration (mg/dL) in women. **B**. Frequency distribution of Lp(a) concentration (mg/dL) in men. Lp(a), lipoprotein (a)
